# A 16-year retrospective study of vascular anomalies in the head and neck region

**DOI:** 10.1186/s13005-023-00376-z

**Published:** 2023-08-01

**Authors:** Bernard Leyman, Dries Govaerts, Jakob Titiaan Dormaar, Jan Meeus, Michel Bila, Ruxandra Coropciuc, Robin Willaert, Constantinus Politis

**Affiliations:** 1 OMFS–IMPATH Research Group, Department of Imaging and Pathology, Faculty of Medicine, Catholic University Leuven, Kapucijnenvoer 33, Leuven, Belgium; 2 Department of Oral and Maxillofacial Surgery, University Hospitals Leuven, Herestraat 49, Leuven, Belgium; 3 Department of Oral and Maxillofacial Surgery, University Hospitals Leuven, Kapucijnenvoer 33, 3000 Leuven, Belgium; 4 Present Address: Radboudumc, Geert Grooteplein Zuid 10, Nijmegen, The Netherlands

**Keywords:** Vascular anomaly, Vascular malformation, Hemangioma, ISSVA

## Abstract

Depending on the diagnostic modality, the classification of vascular anomalies varies and so does the nomenclature. The ‘International Society for the Study of Vascular Anomalies’ (ISSVA) is the most widely accepted classification in the literature and is mainly based on the radiologic and clinical presentation. The aim of this article is to review the clinical practice of diagnosis and treatment of vascular anomalies in the head and neck region in a university hospital, with special focus on the nomenclature.

All patients with a vascular anomaly presenting to the department of oral and maxillofacial surgery were reviewed in a retrospective manner. Nomenclature, diagnostic process, lesion characteristics, treatment and outcome were examined. The lesions were (re)classified according to the ISSVA classification.

A total of 185 patients were identified, of which 12.4% (*n* = 23) had a congenital anomaly. After reclassification, the most common lesions were venous malformations (*n* = 47, 25.4%), followed by lobular capillary hemangiomas (*n* = 17, 9.2%). A group of 39 anomalies could not be further specified. One hundred and one patients (54,6%) received treatment, of which 93 were treated surgically (92,1% of treated patients). Endovascular treatment was considered in 41 patients but applied in only eight. This strict selection led to a low a complication rate.

We provide an overview of the clinical practice in the management of vascular anomalies in a university hospital. The histology report is a source of miscommunication because clinicians use the ISSVA classification, while pathologists use the WHO classification. Every professional involved should be aware of the differences in classification and nomenclature.

**Supplementary Information:**

The online version contains supplementary material available at 10.1186/s13005-023-00376-z.

## Introduction

‘Vascular anomaly’ is an umbrella term for local, structural aberrancies in the vasculature that can affect arteries, veins, capillaries as well as lymphatic vessels [24]. The first classification for vascular anomalies, by Virchow and Wegner, was solely based on histopathological features (Additional file 1) [20]. In 1982 Mulliken and Glowacki classified anomalies based on the endothelial cellular kinetics, coupled with the clinical manifestation and physical findings (Additional file 2) [23, 36]). They divided vascular anomalies into tumors and vascular malformations. This clinically more meaningful classification formed the base for the ‘International Society for the Study of Vascular Anomalies’ (ISSVA) classification. This classification was first introduced in 1996, and is now widely accepted in the literature (Additional file 3) [10]. In clinical practice, the nomenclature does often not match the ISSVA classification [32]. However, a correct terminology is crucial as the vascular anomalies can be diagnosed and treated by a variety of specialists and the various types of anomalies require different treatments [1]. In the past, surgery was the main and sometimes only available treatment modality. In recent years there has been a trend towards more minimally invasive techniques and medication [40].

After a synopsis of the most common types of vascular anomalies, we present an overview of all patients with vascular anomalies who presented at the department of oral and maxillofacial surgery (OMFS) in the university hospitals of Leuven in a 16-year period, focusing on nomenclature, classification, epidemiology, diagnostics, treatment, complications and outcome.

### Tumors

Infantile hemangiomas (IHs) are the most common and best-known vascular tumors. IHs have a characteristic growth pattern: they are absent or merely visible at birth, start growing before the fourth week, stop expanding before the fifth month and involute spontaneously after 6 to 12 months [3, 14]. In 40–50% of the patients, IHs leave residual skin changes, e.g. discolouration, fibro-fatty tissue,… [14]. The standard therapy for IHs is propranolol, which accelerates regression and ultimately reduces residual lesions [39].

Congenital hemangiomas (CHs) are fully developed at birth and have no postnatal proliferative phase. There are two main variants: rapidly involuting CHs start involuting within the first year after birth, while non-involuting CHs never regress [3]. CHs do not respond to pharmacological therapies and thus resection is the treatment of choice [7].

Lobular capillary hemangiomas (LCHs) are common benign vascular tumors with a prevalence of 0.5 to 1% in the general population [9, 34]. They occur mainly in the head-and-neck region and intra-orally, on the gums, tongue, lips and buccal mucosa [9, 38]. The synonym ‘pyogenic granuloma’ is a misnomer because lobular capillary hemangiomas are not associated with pus and do not histologically resemble granulomas [38].

### Vascular malformations

Vascular malformations are always present at birth, whether symptomatic or visible. They never regress spontaneously and grow in relation with the patient. There may be periods of accelerated growth, for example at puberty or pregnancy, due to hormonal changes [15].

Venous, capillary, lymphatic and arteriovenous malformations are simple malformations. Combined malformations consist of two or more types of simple malformations, e.g. capillary-venous malformations, capillary-venous-arteriovenous malformation, … [15]. If a vascular malformation has an arterial component, it is categorized as a high flow malformation. All other malformations have a low flow [15].

#### Venous malformation

A venous malformation (VM) is a soft, non-pulsating, compressible lesion that swells when the central venous pressure rises. The overlying skin or mucosa may be bluish or normal, depending on the depth of the VM [15]. Congestion in the VM can cause chronic pain. Intralesionally formed thrombi evoke a more intense pain [14, 19]. Calcified thrombi, also known as phleboliths, are pathognomonic for VMs [14].

Therapy consists of sclerotherapy, surgical resection, laser-therapy for superficial VMs (Nd:YAG) or a combination of these modalities. Small VMs can be excised if well-defined, with or without prior sclerotherapy, which helps to delineate the lesion and reduces intraoperative blood loss. For ill-defined, infiltrative and/or big lesions, sclerotherapy is the preferred treatment [33]. Laser-therapy is limited by its penetration depth, and thus can only be used for superficial VMs [27].

#### Lymphatic malformation

Lymphatic malformations (LMs) are the most common vascular malformations in the head- and neck region [29]. A local or systemic infection or hemorrhage can cause a temporary growth, which partially regresses once the infection is cleared. A macrocystic LM (individual cysts > 1 cm) feels soft, while the microcystic LM (individual cysts < 1 cm) is rather firm. They are generally painless, unless complicated with infection or intracystic hemorrhage, which turns the overlying skin or mucosa blue. LMs are often accompanied by overgrowth of soft and/or bony tissue [5, 25, 29].

LMs should only be treated if symptomatic or if acute swelling due to infection/hemorrhage could compromise neighboring vital structures (e.g. acutely endangered airway). LMs are difficult to eliminate completely without mutilating the surrounding tissue [37]. Macrocystic LMs can be treated surgically or with sclerotherapy, which are equally effective. Treatment of microcystic LMs is challenging as sclerotherapy is almost impossible and surgery is complicated by the infiltrative nature of microcystic LMs. Treatment with the mTOR inhibitor Sirolimus could address this challenge, as there are promising results [37].

#### Arteriovenous malformation

Arteriovenous malformation (AVM) is an uncommon, dangerous, and difficult-to-treat anomaly. The capillary network between arteries and veins is missing, resulting in abnormal pressure and flow in the first part of the veins, which are dilated. The center of the AVM is called the nidus [6].

The clinical manifestation of an AVM changes as the patient ages and the lesion expands [28].

AVMs can be treated by surgical resection, embolization or (most often) the combination [17]. Because the recurrence rate is high (60–98%), the primary goal of therapy should be symptom relief, prevention of disfigurement and life-threatening situations [6, 17, 30].

#### Capillary malformation

Capillary malformation (CM) is a general term encompassing several types of lesions consisting mainly of capillaries, but with different characteristics and clinical courses. The best known types of CM are the nevus simplex and the nevus flammeus [10].

The nevus simplex, also known as ‘‘angel kiss’’, is a congenital CM most often found in the midline of the face and neck as a pink or bright red, blanchable lesion. The borders are blurred, but the contrast with surrounding tissue enhances with increasing blood pressure or vasodilatation. During the first two years of life, most lesions fade or disappear [13].

The nevus flammeus, also called “port wine stain”, is far less common than the nevus simplex with an incidence of 0,3%. The lesion is caused by a deficiency of the nerves in de the papillary plexus, which causes the capillaries to dilate continuously. The nevus flammeus hypertrophies and darkens with time [11]. In the face, port wine stains follow the distribution of the trigeminal nerve [4].

A CM is usually isolated but can also be part of a syndrome. Sturge-Weber syndrome is characterized by multiple CMs, mostly in the face, the ocular choroid, causing glaucoma, and in the ipsilateral meninges, resulting in epilepsy, stroke and intellectual disability [21].

## Material and methods

All consecutive patients with a vascular anomaly in the head and neck region who consulted the department of oral and maxillofacial surgery from January 2002 to December 2017 were retrospectively reviewed. Patients were included based on the nomenclature of the ISSVA-classification and previously used nomenclature mentioned in the clinical reports. All diagnoses were verified and, if necessary, reclassified according to the ISSVA classification.

Patient records were reviewed for gender, age, disease history, diagnostic techniques, anomaly characteristics, affected anatomical areas, symptoms, treatment, complications and recurrences. Anatomical localization was based on the deep anatomical spaces of the head and neck region as described by Warshafsky et al. [35] AVMs were classified according to the Schobinger classification (Table 1: Schobinger classification) [31]. The nature of treatment was divided into [1] active treatment, [2] conservative treatment, [3] referral and [4] treatment for symptoms originating from already treated or regressed anomalies e.g. correction of an asymmetry. Active treatment modalities consisted of surgery, embolization, sclerotherapy, laser therapy and medical treatment with sirolimus. Surgical treatment distinguished between devascularization and hemodynamic techniques. The aim of devascularization is to completely excise the anomaly, while hemodynamic techniques are intended to reduce the hemodynamic effect and symptoms, without the intention of removing the anomaly altogether [18]. The outcome of the active treatments was scored in three categories: [1] complete regression and resolution of symptoms, [2] partial regression or partial resolution of symptoms and [3] no regression or persistence of symptoms. A chi-squared test was used to test for a difference in outcome (complete, partial or no symptom relief/regression) between hemodynamic and devascularization surgical techniques, between surgery, interventional radiological and laser treatment. A *p* value of < 0.01 was considered significant, with a Bonferroni-correction applied (*p* < 0.05/5).

**Table 1:** Schobinger classification

Stage	Status	Clinical manifestations
Stage I	Quiescent AVM	Hyperemia, cutaneous blush, shunting on doppler
Stage II	Expansive AVM	Pulsations, thrill, bruit, enlargement, tortuous veins
Stage III	Destructive AVM	Ulceration, bleeding or continuous pain
Stage IV	Decompensated AVM	High output heart failure

Complications were classified according to the Clavien-Dindo classification of surgical complications [2].

## Results

### Patient population

A total of 185 patients with a vascular anomaly, including 85 women (46%) and 100 men (54%), consulted the OMFS department of the University Hospitals of Leuven in a 16-year period. The age ranged from 3 months to 94 years and the mean age was 44.2 (SD 23.8) years.

### Diagnostics

Before the first contact, 65 patients (35,1%) had consulted a specialist doctor and 41 patients (22,2%) had undergone previous therapy. Twenty patients (10.8%) who attended the general consultation, were referred to the multidisciplinary consultation for vascular anomalies.

In three quarters of the patient population (*n* = 143; 77.3%), the initial reason for consultation was diagnosis and treatment. In 14 patients (7.6%), the vascular anomaly was an incidental finding and 18 patients (9.7%) requested a second opinion or advise. Six patients (3.2%) requested reconstructive treatment for a defect caused by a vascular anomaly (e.g. resection of fibrofatty tissue).

The most commonly used diagnostic techniques were histopathological examination (44,9%), MRI (29,7%), CT (11,4%) and ultrasound (9,2%). No further investigations were conducted in a quarter of the patients. The list of all technical investigations can be found in Additional file 4.

### Lesion characteristics

Twenty-three lesions (12.4%) were present at birth and 123 lesions (66.5%) developed later in life at a mean age of 41.8 years (SD 22,9 years). In 39 lesions (21.1%), the time of onset was not recorded.

Venous malformations were diagnosed most frequently (47 patients, 25.4%), followed by lobular capillary hemangiomas (17 patients, 9.2%), arteriovenous malformations (13 patients, 7.0%) and combined malformations (10 patients, 5.4%) (Table 2).

**Table 2:** Frequency of the different vascular anomalies in the study population

Vascular tumors			Vascular malformations						Not specified		
	N	%^a^	**Low flow**	N	%^a^	**High flow**	N	%^a^		N	%^a^
LCH	17	9.2	VM	47	25.4	AVM	13	7.0	Vascular anomaly NOS	39	21.1
IH	1	0.5	LM	3	1.6	Combined	3	1.6			
CH	1	0.5	CM	3	1.6						
KHE	1	0.5	Combined	7	3.8						
IPEH	1	0.5	Sturge Weber	2	1.1						
			Vascular malformation NOS				47	25.4			
Total	19	10.3	Total				125	67.6	Total of all anomalies	185	100

^a^Percentage of the total number of anomalies (*N* = 185); *LCH* lobular capillary hemangioma, *IH* infantile hemangioma, *CH* congenital hemangioma, *KHE* kaposiform hemangioendothelioma, *IPEH* intravascular papillary endothelial hyperplasia, *NOS* not otherwise specified, *VM* venous malformation, *AVM* arterio-venous malformation, *LM* lymphatic malformation, *CM* capillary malformation

Three AVMs were in Schobinger stage I, seven in stage II and three in stage III (none in stage IV). Forty-seven vascular malformations (25.4%) were not further subclassified and 39 vascular anomalies (21,1%) could not be specified according to the ISSVA classification based on the clinical records. Two patients had Sturge Weber syndrome and one patient had a Kaposiform hemangioendothelioma, a locally aggressive tumor. Another patient had a Masson’s tumor, which is a benign tumor.

Most lesions were unifocal (*n* = 146; 78.9%) and half of the anomalies had diffuse borders (*n* = 97; 52.4%). For 35 lesions (18.9%) there was no information on demarcation.

The lips were most frequently affected (*n* = 72; 38.9%), followed by the tongue (*n* = 46; 24.9%), buccal space (*n* = 36; 19.5%), sublingual space (*n* = 13; 7.0%), masticatory space and gums (both 12 lesions; 6.5%). The complete list of affected anatomical regions can be found in Additional file 5.

Most frequently mentioned symptoms were discomfort of the bulky lesion (*n* = 34; 18.4%), unpleasant aesthetics (*n* = 25; 13.5%), pain (*n* = 24; 13.0%) and bleeding (*n* = 21; 11.4%). One-third of patients were asymptomatic (*n* = 66, 35.7%). A list of all symptoms can be found Additional file 6.

### Treatment and outcome

A total of 101 patients (54.6%) received active treatment and 12 of them (6.5%) received multiple therapies. Vascular anomalies were surgically removed with devascularization techniques in 90 surgical procedures in 83 patients (44.9%) and with hemodynamic techniques in 16 procedures in 10 patients (5.4%). Devascularization techniques resulted in complete symptom relief in 84.4% of procedures, while hemodynamic techniques led to complete symptom relief in 43.8% (*p* = 0.0003). A minority were treated with laser therapy (*n* = 6, 3.2%), sclerotherapy (*n* = 5, 2.7%), embolization (*n* = 3, 1.6%) or sirolimus (*n* = 1, 0.5%). The outcome of surgery was better than interventional radiological therapy (*p* = 0.000003), but not better than laser therapy (*p* = 0.31). Thirteen patients (7.0%) were informed about the treatment options but did not desire therapy. A quarter of patients (*n* = 50, 27.0%) were treated conservatively. Thirteen patients (7.0%) were referred to other departments for treatment and seven patients (3.8%) were lost to follow-up before treatment started. All therapies and outcomes are listed in Additional file 7.

In 57 cases (30.8%), treatment or treatment planning took place through multidisciplinary collaboration. Interventional radiology was consulted most often (41 cases, 22.2%) to assess the feasibility of embolization or sclerotherapy. Other involved disciplines are listed in Additional file 8.

Complications were reported in 18 patients (17.8%) of the 101 patients who received active treatment (Table 3). The most common complications were Clavien-Dindo grade I or II: impaired wound healing (*n* = 5, gr. I), wound infection (*n* = 4, gr. I), temporary local hypoesthesia (*n* = 7, gr. II) and permanent local hypoesthesia (*n* = 3, gr. II). During two operations, the vascular anomaly bled excessively, necessitating admission to intensive care (grade IV).

**Table 3:** Incidence of all complications after active treatment, according to Clavien-Dindo classification

Complications	I	II	IIIa	IIIb	IVa	IVB	V	N (%^a^)
Bacterial / fungal infection	7	-	-	-	-	-	-	7 (6.9%)
Temporary hypoesthesia	-	4	-	-	-	-	-	4 (4.0%)
Permanent hypoesthesia	-	3	-	-	-	-	-	3 (3.0%)
Impaired healing	5	-	-	-	-	-	-	5 (5.0%)
Prolonged reduced mouth opening	2	-	-	-	-	-	-	2 (2.0%)
Keloid scarring	2	-	-	-	-	-	-	2 (2.0%)
Excessive intraoperative bleeding	-	-	-	-	2	-	-	2 (2.0%)
Persistent pain (not due to infection)	1	-	-	-	-	–	-	1 (1.0%)
Facial nerve dysfunction	-	1	-	-	-	-	-	1 (1.0%)
Diarrhea in association with Sirolimus	-	-	-	-	-	-	-	1 (1.0%)
Sialocele	-	1	-	-	-	-	-	1 (1.0%)
Dysphagia	1	-	-	-	-	-	-	1 (1.0%)

^a^Percentage of patients receiving active treatment (surgery, sclerotherapy, embolization, laser therapy and sirolimus) (*n* = 101)

In nine of the actively treated patients (8.9%), the vascular anomaly recurred: four with an AVM, three with a VM and two with an LCH. Three patients had multiple recurrences, ranging from two and four. Recurrence occurred after embolization of AVMs in three patients and after surgical therapy in the remaining six patients. All recurrences were treated surgically, with or without preoperative embolization. Another twenty-two patients (11.9%) reported a recurrent anomaly at initial consultation. All these lesions were treated again at UZ Leuven with no new recurrences.

## Discussion

### Diagnosis and classification

IH account for 85% of vascular tumors in the general population and tumors account for one third of the vascular anomalies [8]. In this study population, only 10.3% (*n* = 19) of anomalies were vascular tumors and only one lesion was identified as an IH. Because most IH are treated with propranolol by pediatricians at a young age, few patients visit the OMFS department. Surgical indications are limited to complicated IH and large residual lesions [39].

The histopathology report proved to be a major source of confusion regarding the nomenclature. Pathologists use the WHO classification for head and neck tumors, while radiologists and clinicians use the ISSVA-classification. The WHO classification does not distinguish between hemangiomas and vascular malformations because it is difficult to do so, based on histology alone. They divide benign vascular lesions based on anatomy, constituent vessels, border characteristics and genetic alterations [16].

We found that in clinical records, the nomenclature for the diagnosis often contained terms from previous classifications or the WHO classification. We therefore reclassified the lesions according to the ISSVA-classification to the best of our ability, considering the retrospective character of the study. For the reclassification, radiologic imaging was a backbone. MRI and ultrasound can distinguish between lesions with low and high blood flow and, as with CT, identify feeding and draining vessels. Thirty-two lesions were named ‘cavernous hemangioma’. This term was abandoned by the ISSVA. It was used to describe an IH with a deep component in the (sub)cutis and in histopathological reports it defines a venous-type vascular lesion with large lumens, thin walls and flattened endothelial cells [9, 26]. The suffix ‘-oma’ is used to describe a neoplasm [22]. However, reclassification of cavernous hemangiomas according to the ISSVA classification reveals 88% to 100% to be a vascular malformations [12, 26]. Therefore ‘cavernous hemangioma’ is a misnomer and should be avoided in clinical reports. In this study 20 cavernous hemangiomas were reclassified as venous malformations based on anamnesis, radiological, histopathological and clinical examination. The remaining 12 cavernous hemangiomas that had not been examined histopathologically were classified as vascular malformations (6.5%) without further specification.

A group of 39 anomalies (21.1%) could not be reclassified with certainty according to the ISSVA classification. Most of these lesions were termed ‘hemangiomas’, which is often used as an abbreviation for IH [3]. Because these ‘hemangiomas’ had developed later in life, they could not, by definition, be infantile or congenital hemangiomas.

Due to retrospective reclassification, the prevalence of the different types of anomalies in the study population did not always match the prevalences in the general population. Only VMs were reported equal (37.6% and 36.8% of the malformations, respectively) [8]. All other types of malformations were diagnosed less frequently than in the general population, especially LMs (2.4% vs. 28.3% respectively) [8].

In two patients the correct diagnosis was delayed. One sublingual lesion was initially diagnosed and treated as a hemorrhagic ranula. The correct diagnosis was made after the lesion recurred 5 years later. In another patient, phleboliths of a VM were initially diagnosed and treated as sialoliths.

### Treatment

Surgery was the main treatment method: 50% of all patients (*n* = 93) or 92% of all actively treated patients underwent surgery. In 81 patients, surgery was a monotherapy, mostly excisional biopsies of small lesions. Surgery was found to result in fewer persisting symptoms after treatment compared to the other active treatments. This may be partly explained by the fact that mainly small lesions were considered for surgery (often excisional biopsies). While for the larger and more complex lesions, interventional treatments were considered to avoid large anatomical defects, damage to neighboring anatomical structures and the risk of intraoperative excessive bleeding. Compared with surgery, they have the disadvantage of only reducing the volume of the lesion but not completely eliminating it.

Treatment of AVMs is challenging due to the high recurrence rate and high intraoperative bleeding risk. In this cohort, four of the eleven actively treated AVMs recurred. All three embolization-treated AVM patients showed recurrence, which is consistent with the 98% recurrence rate in the literature [17].

With one of seven surgically treated AVMs relapsing, the recurrence rate is lower than the 81% reported in literature [17]. Partial resection of a large AVM has a high risk of recurrence, extensive intraoperative bleeding and acute worsening of the disease [1, 6]. This advocates for a more radical approach, with flap reconstruction if necessary. In this study, two patients required reconstruction with a radial forearm flap and remained disease free afterwards. One AVM patient had a history of multiple embolization procedures and recurrences with spontaneous bleedings. Another embolization was not safe, thus the external carotid artery was ligated to stop bleeding. The decision to clip the external carotid artery was inevitable because of the size of the bone lesion and because all distant, smaller arteries had been ligated in previous surgeries. Clipping the major feeding arteries does not have a good long-term outcome. It can only be considered as a salvage therapy to stop a life-threatening bleeding [40].

CMs were rarely seen in the OMFS practice and were never operated. The primary therapy is pulsed dye laser therapy and surgery is only indicated to correct underlying tissue deformities, e.g. associated overgrowth of soft or bony tissue [1]. All CM patients were referred to the dermatology department for treatment.

In the last three years of the study, there was a tendency to refer every patient to the multidisciplinary consultation. The team consists of doctors from the departments of vascular and plastic surgery, hematology, pediatric hematology, cardiology, radiology and OMFS. Working in a multidisciplinary team makes it easy to discuss the various treatment options, which are often carried out by different disciplines. Interventional radiologists were consulted most often. They screened 41 patients, but proceeded to endovascular treatment in only eight patients (7.9% of actively treated patients). The main reason to abstain from endovascular treatment was the risk of necrosis in superficial lesions, especially in the esthetic zones. This strict indication selection resulted in a low complication rate. No complications occurred in five sclerotherapy -treated patients. In the 10 embolization sessions performed in three patients, one procedure was complicated with dysphagia due to postoperative swelling.

## Conclusion

Many classifications have been used for vascular anomalies. Now, the ISSVA classification is the preferred system for clinicians and radiologists. Pathologists on the other hand, use the WHO classification, which also involves a different nomenclature. Until a unified classification is available, clinicians can best use the histology report in the light of the ISSVA classification to confirm, reject or specify the diagnosis. The terms “cavernous hemangioma” and “hemangioma” are problematic and should be interpreted with caution.

More minimally invasive treatments have become available, including medication. As a result of ß-blocker administration for IHs at a young age, only 10% of anomalies at OMFS are vascular tumors. Embolization and sclerotherapy are viable and safe treatment options, provided strict patient selection. Despite the emergence of alternative treatments, surgery remains the main treatment modality. Devascularization techniques are preferred to hemodynamic techniques as they minimize the risk of recurrent symptoms and excessive intraoperative bleeding.

## Supplementary Information

**Additional file 1.** Classificationfor vascular anomalies, by Virchow and Wegner. Classification of vascularlesions according to Virchow, published in 1863 and classification of lymphaticlesions, according to Wegner, published in 1977. The third column describes thehistological image that can be found for each type of lesion.

**Additional file 2.** Classificationfor vascular anomalies, by Mulliken and Glowaki.Classification of vascular lesions according to Mulliken and Glowaki,published in 1982. The vascular anomalies are devided in tumors (hemangiomas,first column) and malformations (second column). The hemangiomas have aproliferating and involuting phase. The malformations are divided according totheir composing vessels.

**Additional file 3.** ISSVAclassification. Table shows the classification of the vascular anomaliesaccording to the “International Society for the Study of Vascular Anomalies”(ISSVA) as presented in 2018.

**Additional file 4.** Diagnostictechniques. List of the additional techniques, used to diagnose the vascularanomaly, as well as the number of times each technique was used (N and %).

**Additional file 5.** Anatomicalregions affected by the vascular anomaly. List of anatomical regions affectedby the vascular anomaly, as well as the number of times each region wasaffected (N and %).

**Additional file 6.** Symptomsat registration or before the start of treatment. List of symptoms atregistration or before the start of treatment, as well as the number of timeseach symptom was registered (N and %).

**Additional file 7.** Performedtreatments and outcomes. List of performed treatments (N and %) and theiroutcomes (N).

**Additional file 8.** Departmentsinvolved in treatment(planning). List of departments involved in treatment (planning),as well as the number of times each department was involved (N and %).

## Data Availability

The dataset generated during the current study is available from the corresponding author on reasonable request.

## Abbreviations

ISSVA: International Society for the Study of Vascular Anomalies
OMFS: Department of oral and maxillofacial surgery
AVM: Arterio-venous malformation
VM: Venous malformation
LCH: Lobular capillary hemangioma
IH: Infantile hemangioma
CH: Congenital hemangioma
KHE: Kaposiform hemangioendothelioma
IPEH: Intravascular papillary endothelial hyperplasia
NOS: Not otherwise specified
LM: Lymphatic malformation
CM: Capillary malformation
